# Impact of tumor necrosis factor inhibitors and methotrexate on diabetes mellitus among patients with inflammatory arthritis

**DOI:** 10.1186/s41927-020-00138-3

**Published:** 2020-09-02

**Authors:** Santhi Mantravadi, Michael George, Colleen Brensinger, Min Du, Joshua F. Baker, Alexis Ogdie

**Affiliations:** 1 Department of Pharmacology and Experimental Therapeutics, Thomas Jefferson University, 132 South 10th Street, 1170 Main Building, Philadelphia, PA 19107-5244 USA; 2 Department of Medicine, Division of Rheumatology, Perelman School of Medicine, University of Pennsylvania, White Building Rm 5023, 3400 Spruce St, Philadelphia, PA 19104 USA; 3 Department of Biostatistics, Epidemiology, and Informatics, Division of Rheumatology, Perelman School of Medicine, University of Pennsylvania, 423 Guardian Dr, Philadelphia, PA 19104 USA

**Keywords:** Psoriatic arthritis, Rheumatoid arthritis, Ankylosing spondylitis, Diabetes mellitus, Outcomes, Epidemiology

## Abstract

**Background:**

To determine whether initiation of a tumor necrosis factor inhibitor (TNFi) or methotrexate improves hemoglobin A1c in patients with psoriatic arthritis (PsA), rheumatoid arthritis (RA), or ankylosing spondylitis (AS) who also have diabetes mellitus (DM).

**Methods:**

A retrospective cohort study was conducted in Optum’s de-identified Clinformatics® Data Mart Database, an administrative claims database, using data from 2000 to 2014. Patients with PsA, RA, or AS, with DM (defined by ICD-9-CM codes) and/or HbA1c ≥7%, who newly initiated either a TNFi, MTX, or metformin (positive control) were identified. The change in HbA1c after drug initiation was calculated. Statistical differences in the change in HbA1c between drugs were assessed using the Wilcoxon rank sum test and linear regression models adjusting for potential confounders.

**Results:**

Among 10,389 drug initiations in 9541 patients with PsA, RA, or AS, and available HbA1c values, HbA1c was ≥7 at baseline in 254 (35%) TNFi initiations, 361(37%) MTX initiations, and 2144 (50%) metformin initiations. Median HbA1c change was − 0.35 (IQR -1.10, 0.30) after TNFi initiation, − 0.40 (IQR -1.20, 0.30) after MTX initiation, and − 0.80 (IQR -1.60, − 0.10) after metformin initiation. In adjusted analyses, TNFi initiators had less of a decrease in HbA1c compared to MTX initiators (β 0.22, 95% CI: 0.004, 0.43), *p* = 0.046. Metformin initiators had a significantly greater decrease in HbA1c than MTX, β − 0.38 (95% CI: − 0.52, − 0.23), *p* < 0.001. Glucocorticoid use was not accounted for in the models.

**Conclusion:**

HbA1c decreased with TNFi initiation or MTX initiation. Reductions in HbA1c after initiation of a TNFi or MTX are about half (**~** 0.4 units) the decrease observed after initiation of metformin.

## Background

RA, PsA, and AS are chronic debilitating inflammatory joint diseases associated with significant comorbidities such as DM, cardiovascular disease, and depression [1–4]. Incidence and prevalence of DM is increased in patients with RA, PsA and AS patients, affecting approximately 10% or more of patients with IA [1, 3, 5–8].

Tumor necrosis factor (TNF)-α, an inflammatory cytokine, is a key player in the pathogenesis of RA, PsA, AS, and type II DM. Elevated local and circulating levels of TNF-α in RA, PsA, and AS contribute to the inflammatory and structural changes in these inflammatory conditions [9–11]. In type II diabetes, a chronic inflammatory state exists, marked by increased cytokines such as TNF-α, IL-1, and IL-6 [12]. Patients with inflammatory arthritis were also found to have greater insulin resistance compared to controls, and this was particularly true for PsA [13]. TNF-α has been found to promote insulin resistance [14, 15] and disrupt insulin signaling [16, 17]. Thus, it may be presumed that modulating this pathway in IA patients with diabetes may improve insulin sensitivity. In several studies, the Homeostatic Model Assessment for Insulin Resistance (HOMA-IR index), which reflects insulin resistance, decreased with TNFi therapy in RA patients [18–21]. Additionally, another study suggested that infliximab treatment in RA and AS patients may improve insulin sensitivity in patients with high insulin resistance [22]. This study did not compare TNFi use to other common DMARDs.

Little is known about the role of MTX in insulin resistance and existing studies have conflicting messages. One study observed decreases in HbA1c in RA and PsA patients without diabetes taking MTX [23]. In addition, the prevalence of metabolic syndrome in patients with RA was found to be lower among patients using methotrexate, though this data may be confounded by the steatohepatitis leading to discontinuation of methotrexate [24]. However, in a large retrospective study of patients with RA or psoriasis, use of a TNFi was associated with a decreased risk for diabetes whereas use of methotrexate was not [25]. In other studies, significant decreases were not seen in the HOMA-IR index with initiation of MTX [23, 24, 26].

Overall, little is known about how TNFi and MTX impact HbA1c in patients with IA and diabetes. The goal of this study was to determine whether HbA1c improves in DM patients with RA, PsA, or AS initiating a TNFi compared to patients initiating MTX in a large real-world cohort. We hypothesized that patients using TNFi would have a greater reduction in HbA1c than patients initiating methotrexate based on the pathophysiologic role of TNF in diabetes.

## Methods

### Study design and data source

A retrospective cohort study was conducted in Clinformatics™ Data Mart (OptumInsight, Eden, Prairie, MN), a de-identified administrative claims database in the United States that includes demographic data, prescription drug use, diagnostic codes, medical claims history, and laboratory values (approximately 10% of patients) for approximately 13 million beneficiaries. We identified patients with RA, PsA, or AS with an HbA1c ≥ 7 and examined change in HbA1c among new initiators of a TNFi (etanercept, adalimumab, certolizumab, golimumab, or infliximab), MTX, or metformin (positive control). In addition, we studied all patients with IA and DM fulfilling criteria defined by ICD-9-CM codes and with a baseline HbA1c regardless of the value (“DM criteria cohort”).

### Study time period

**Fig. 1:**
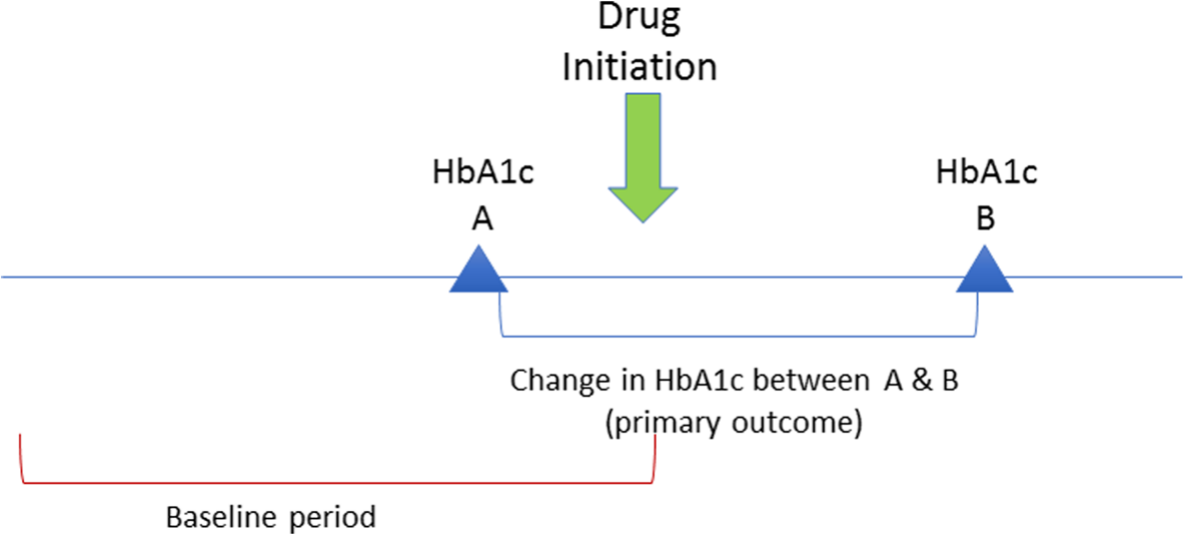
Study Design and Study Time Period. Start date is the date of drug initiation (TNFi, MTX, or metformin). A baseline period prior to the drug start date was required to capture any potential confounders. Baseline HbA1c (HbA1c A) must have occurred within the 6 months prior to medication initiation. Follow up HbA1C (HbA1c B) was at least 3 months from the prior value and three to 12 months from initiating the medication. Change in HbA1c value from before to after drug initiation was reported for TNFi, MTX, and metformin users (primary outcome). Abbreviations: HbA1c = hemoglobin A1c; MTX = methotrexate; TNFi = tumor necrosis factor inhibitor

Data from 2000 to 2014 were included. The index date was the date of TNFi, MTX, or metformin initiation. A baseline period any time prior to the index date was required to capture potential confounders [27–29]. All patients were required to have one HbA1c in the 6 months prior to and one HbA1c in the 3-12 months after drug initiation. Follow up HbA1C was at least 3 months from the prior value and 3 months from initiating the medication. (Fig. 1).

### Study population

Patients ≥18 years of age with at least one diagnosis code for PsA (ICD-9-CM 696.0), or RA (ICD-9-CM 714.0–714.33), or AS (ICD-9-CM 720.0) prior to therapy initiation, and a prescription for a disease-modifying antirheumatic drug (DMARD) were included in the cohort. Studies have shown a higher PPV when diagnosis codes are combined with DMARD prescriptions to identify PsA, RA, and AS patients in health care utilization databases [30–35]. In a sensitivity analysis, we allowed patients to acquire the code for IA at any point during follow up. Among patients with IA, we identified diabetes using diagnosis codes (ICD-9-CM 249.xx, 250.xx, 357.2, 362.01–06, 366.41) [36, 37]. The primary analysis included patients with a HbA1c ≥ 7. In a secondary analysis, we included patients who met “DM criteria.” These patients were required to have one inpatient code or two outpatient codes at any point in the database (PPV approximately 90%) [38]. Similar definitions have been used in prior studies [39, 40]. The distinction was not made between Type I and Type II DM, but given the age of the cohort, the patients are likely to predominantly have Type II DM [41].

### Exposures

The primary exposures of interest were new initiations of TNFi (etanercept, adalimumab, certolizumab, golimumab, or infliximab) and MTX (oral or subcutaneous). Medications were identified using National Drug Codes (NDC). We used a new user design in which patients were initiating a new TNFi or methotrexate and had not previously had a prescription for the therapy initiated. Patients may have had a prior exposure to a different TNFi. Patients were required to have at least 12 months in the dataset prior to therapy initiation to evaluate prior exposures and to ensure new drug start. We additionally included metformin as a “positive control” using similar definitions.

### Outcome definition

The outcome of interest was the absolute change in HbA1c after initiation of a TNFi, MTX, or metformin.

### Covariates

We examined baseline demographics, comorbidities, and medications, and their impact on HbA1c change. These included age, sex, anemia, angina, aortic aneurysm, asthma, anxiety, atrial fibrillation, baseline diabetes medications, baseline methotrexate, baseline TNFi, bipolar disorder, cancer, cardiomyopathy, chronic kidney disease, chronic obstructive pulmonary disease, connective tissue disease, cerebrovascular disease, congestive heart failure, coronary artery disease, degenerative disc disease, dementia, depression, diabetic retinopathy, giant cell arteritis, hypertension, hypothyroidism, inflammatory bowel disease, lung disease, metabolic syndrome, mixed connective tissue disease, myocardial infarction, obesity, obstructive sleep apnea, osteoarthritis, peptic ulcer disease, peripheral arterial disease, peripheral vascular disease, pulmonary embolism, polymyalgia rheumatica, pregnancy, psoriasis, other psychiatric disorders, rheumatoid arthritis-lung, SICCA syndrome, sleep disorder, systemic lupus erythematous, systemic sclerosis, and uveitis. These covariates were defined by plan demographic information, ICD-9-CM codes, and NDC codes. We specifically did not adjust for glucocorticoids as glucocorticoids may be on the causal pathway.

### Statistical analysis

All statistical analysis was performed using STATA 15.0 (StataCorp, College Station, TX, USA). The primary analysis (among patients with HbA1c ≥ 7) and secondary analysis (DM criteria cohort) were performed in the same manner. Median HbA1c change was calculated (given non-normal distribution), and the Wilcoxon signed-rank test was used to assess the difference in A1c change between baseline A1c change and A1c change after initiation of the medication. The Wilxocon rank sum test was then used to assess the unadjusted statistical differences between the medication groups.

Univariable and multivariable linear regression models were used to determine the relative differences in HbA1c change using MTX as the reference and adjusted for potential confounders. Variables examined as confounders include age, sex, baseline HbA1c, baseline DM medications, baseline MTX use (for patients on TNFi), and baseline comorbidities. Estimates from regression models considered clustering on patients to account for the presence of multiple new drug initiations per patient. In sensitivity analyses, we only allowed patients to contribute one therapy initiation and we included calendar year in the multivariable models.

### Ethics approval

This study was considered exempt by University of Pennsylvania Institutional Review Board.

## Results

**Fig. 2:**
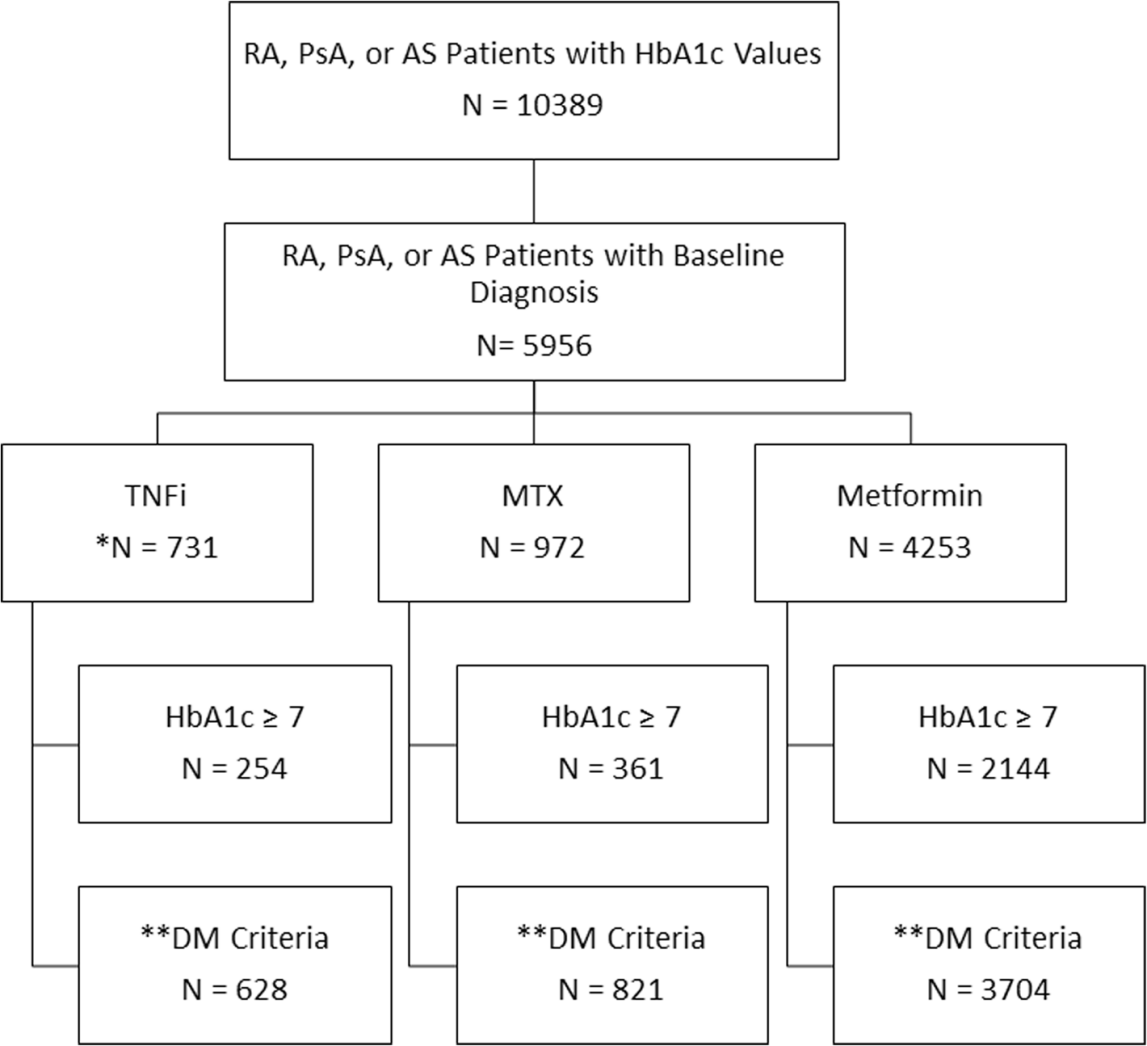
Flow Diagram: Derivation of the Cohorts. The HbA1c ≥ 7 & DM criteria cohorts are not mutually exclusive. *Patients were allowed to enter the TNFi group multiple times with each initiation of a different TNFi. **DM criteria cohort: all patients with DM fulfilling criteria by ICD-9-CM codes and with a baseline HbA1c regardless of the value of the HbA1c. *Abbreviations*: AS = ankylosing spondylitis; DM = diabetes mellitus; HbA1c = hemoglobin A1c; MTX = methotrexate; PsA = psoriatic arthritis; RA = rheumatoid arthritis; TNFi = tumor necrosis factor inhibitor

A total of 10,389 drug initiations in 9541 patients with PsA, RA, or AS, and HbA1c values available were identified: 690, 3681, and 1585 drug initiations with PsA, RA, and AS diagnoses respectively in the baseline period were identified (disease groups are not mutually exclusive, Fig. 2). Of these, there were 731 TNFi initiations, 972 MTX initiations, and 4253 metformin initiations. HbA1c was ≥7 before treatment initiation (the HBA1c ≥ 7 cohort) in 254 (35%) TNFi users, 361 (37%) MTX users, and 2144 (50%) metformin users. DM criteria was fulfilled with no restriction on baseline HbA1c value (the DM criteria cohort) in 628 (86%) TNFi initiations, 821 (84%) MTX initiations, and 3704 (87%) metformin initiations.

Baseline characteristics of the populations are shown in Table 1. In the HbA1c ≥ 7 cohort, the mean age of the TNFi group, MTX group, and metformin group was approximately 45 years. All three groups were predominantly female (60–68%). The mean number of days between the baseline HbA1c and follow-up HbA1c was similar across all three groups (201–241 days). There was a high prevalence of dyslipidemia and hypertension, as expected in a diabetes cohort [42–44]. More patients in the elevated HbA1c group were on diabetes medications in the baseline period than those in the DM criteria cohort (TNFi 91% vs 76%, MTX 88% vs 72%). Baseline characteristics were similar between the primary cohort (HbA1c ≥ 7) and the DM criteria cohort (Suppl Table 1).

**Table 1:** Baseline Characteristics: HbA1c ≥ 7 Cohort

	MTX (*N* = 361)	TNFi (*N* = 254)	Metformin (*N* = 2144)	SMD TNF vs MTX	SMD Met vs MTX
Mean age, years (SD)^a^	45 (3)	45 (3)	45 (3)	0	0
Male sex (%)^b^	37%	32%	40%	−0.15	0.08
Baseline HbA1c	8.29	8.31	8.41	−0.02	0.07
Comorbidities, N (%)					
Anemia	55(15%)	29 (11%)	232 (11%)	−0.11	−0.11
Angina	59 (16%)	28 (11%)	227 (11%)	−0.15	− 0.15
Anxiety	35 (10%)	39 (15%)	375 (17%)	0.15	0.21
Asthma	73 (20%)	48 (19%)	388 (18%)	−0.03	− 0.05
CAD	121 (34%)	60 (24%)	544 (25%)	−0.22	−0.20
CHF	49 (14%)	28 (11%)	251 (12%)	−1.46	−0.16
CKD	103 (29%)	56 (22%)	360 (17%)	−0.28	−0.52
COPD	56 (16%)	48 (19%)	393 (18%)	0.08	0.05
Cardiomyopathy	19 (5%)	9 (4%)	88 (4%)	−0.25	−0.25
Other CTD	21 (6%)	12 (5%)	67 (3%)	0.20	0.71
Depression	71 (20%)	65 (26%)	503 (23%)	0.26	0.14
DM retinopathy	64 (18%)	42 (17%)	29 (1%)	−0.06	−1.37
Hypertension	316 (88%)	217 (85%)	1807 (84%)	−0.03	−0.05
Hypothyroidism	112 (31%)	88 (35%)	671 (31%)	0.12	0.00
Dyslipidemia	312 (86%)	225 (89%)	1825 (85%)	0.03	−0.01
Inflammatory bowel disease	9 (2%)	12 (5%)	44 (2%)	0.90	0.00
Liver disease	88 (24%)	64 (25%)	408 (19%)	0.04	−0.24
Myocardial infarction	23 (6%)	8 (3%)	36 (2%)	−0.71	−1.00
Obesity	98 (27%)	72 (28%)	629 (29%)	0.04	0.07
Psoriasis	43 (12%)	81 (32%)	154 (7%)	0.84	−0.54
Baseline diabetes medications, N (%)	316 (88%)	232 (91%)	2144 (100%)	0.03	0.13
Baseline MTX, N (%)	–	128 (50%)	274 (13%)	n/a	n/a
Baseline TNFi, N (%)	36 (10%)	84 (33%)	214 (10%)	0.96	0.00
Baseline Steroids^c^, N (%)	152 (42%)	100 (39%)	394 (18%)	−0.07	−0.75
Average baseline glucocorticoid dose, mean (SD)	6 (7)	6 (6)	5(7)	0	0.14
Duration btwn baseline and f/up HbA1c, mean # of days (SD)	241 (83)	237 (84)	201 (79)	0.05	−0.49
Duration btwn med start date and f/up HbA1c, mean # of days (SD)	173 (66)	166 (65)	168(66)	−0.11	−0.08

*N* number of observations

TNFi, MTX, and metformin groups are not mutually exclusive

^a^In the metformin group, one observation is missing for age

^b^In the metformin group, two unknown observations for sex were changed to missing

^c^Six-month baseline period

Abbreviations: *CAD* Coronary artery disease, *CKD* Chronic kidney disease, *CTD* Connective tissue disease (ICD9 code 710.9, not inclusive of SLE, sicca, or scleroderma), *COPD* Chronic obstructive pulmonary disease, *CHF* Congestive heart failure, *DM* Diabetes mellitus, *HbA1c* Hemoglobin A1c, *MTX* Methotrexate, *SMD* Standardized mean difference, *TNFi* Tumor necrosis factor inhibitor

In the HbA1c ≥ 7 cohort, the median HbA1c change was − 0.35 (IQR -1.10, 0.30) after TNFi initiation, − 0.40 (IQR -1.20, 0.30) after MTX initiation, and − 0.80 (IQR -1.60, − 0.10) after metformin initiation. In unadjusted analysis, there was no significant difference in the median HbA1c change between TNFi and MTX (*p = 0.46*). There was a statistically significant difference in the median HbA1c change between TNFi and metformin *(p < 0.001)* and between MTX and metformin (*p < 0.001)*.

**Fig. 3:**
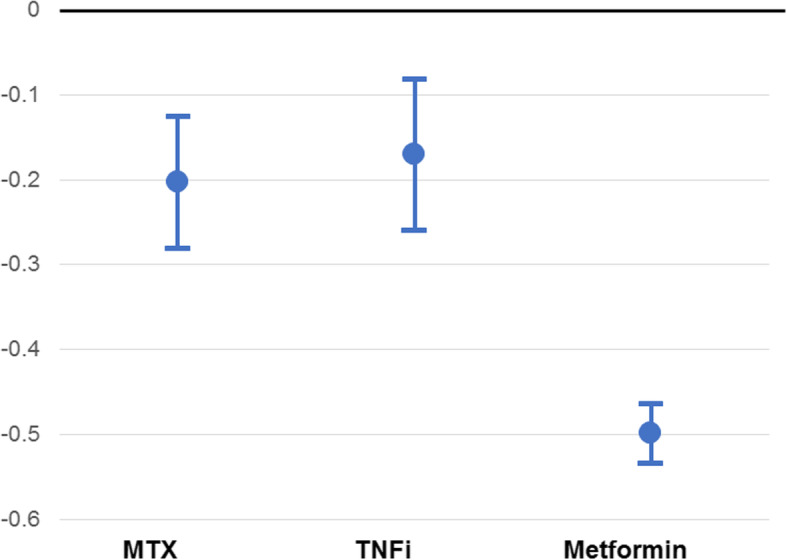
Predicted Change in HbA1c in baseline A1c ≥ 7 cohort after TNFi, MTX, or Metformin Initiation. Predicted change in HbA1c from linear regression models adjusted for age, sex, baseline HbA1c, baseline DM medications, baseline MTX use (for patients on TNFi), and baseline comorbidities. Abbreviations: DM = diabetes mellitus; HbA1c = hemoglobin A1c; MTX = methotrexate; TNFi = tumor necrosis factor inhibitor

In the cohort with HbA1c ≥ 7, after adjustment for age, sex, baseline HbA1c, and baseline comorbidities, metformin initiators had a significantly greater decrease in HbA1c than MTX initiators, β − 0.38 (95%CI: − 0.52, − 0.23), *p < 0.001* (Table 2, Fig. 3, Suppl Figure 1). TNFi initiators had less of a decline in HbA1c compared to MTX initiators (resulting in a positive beta coefficient: β 0.22 (95%CI: 0.004, 0.43), *p = 0.046*.

**Table 2:** Associations Between Treatment Initiation and Change in HbA1c in Patients with Baseline HbA1c ≥ 7

Variable	Univariable Model		Multivariable Model	
	β^a^	95% CI	β^a^	95%CI
Treatment initiation				
Methotrexate	Ref	–	Ref	–
TNFi	0.20	−0.04, 0.43	0.22	0.004, 0.43
Metformin	−0.48	−0.66, − 0.30	−0.38	− 0.52, − 0.23
Age (years)	0.01	− 0.01, 0.03	−0.002	− 0.02, 0.01
Sex (female)	−0.25	− 0.38, − 0.12	−0.20	− 0.30, − 0.10
Baseline HbA1c	−0.68	− 0.73, − 0.63	−0.68	− 0.73, − 0.62
Atrial Fibrillation	0.24	0.03, 0.45		
CAD	0.20	0.06, 0.33		
Cardiomyopathy	0.35	0.02, 0.69	0.30	0.03, 0.57
Other CTD	−0.42	−0.76, −0.08	−0.43	− 0.64, − 0.22
CVD	0.24	0.06, 0.41		
Dementia	0.50	0.19, 0.82		
Hypertension	0.25	0.05, 0.45		
Dyslipidemia	0.28	0.09, 0.46	0.15	0.01, 0.29
Myocardial Infarction	0.66	0.26, 1.06	0.46	0.07, 0.85
PVD	0.28	0.08, 0.48		
Pulmonary Embolism	0.58	0.14, 1.03		

^a^Beta-coefficients are interpreted as the mean difference in the outcome (HbA1c) in the target group (TNF or Metformin) minus the reference group (MTX)

Variables tested that were not significant at the univariable stage were not included in this table: The following variables are not significant at the univariable stage: anemia, angina, anxiety, aortic aneurysm, asthma, baseline diabetes medications, baseline methotrexate, baseline TNFi, bipolar disorder, cancer, chronic kidney disease, chronic obstructive pulmonary disease, congestive heart failure, coronary heart disease, degenerative disc disease, depression, diabetic retinopathy, giant cell arteritis, hypothyroidism, inflammatory bowel disease, liver disease, lung disease, metabolic syndrome, mixed connective tissue disease, obesity, obstructive sleep apnea, osteoarthritis, peptic ulcer disease, peripheral arterial disease, polymyalgia rheumatica, pregnancy, psoriasis, other psychiatric disorders, rheumatoid arthritis-lung, SICCA syndrome, sleep disorder, systemic lupus erythematous, systemic sclerosis, and uveitis

Abbreviations: *CAD* Coronary artery disease, *CTD* Connective tissue disease (ICD9 code 710.9, not inclusive of SLE, sicca, or scleroderma), *CVD* Cerebrovascular disease, *HbA1c* Hemoglobin A1c, *PVD* Peripheral vascular disease, *TNFi* Tumor necrosis factor inhibitor

When conducting the same analyses in the broader DM criteria cohort, the median HbA1c change was 0 (IQR -0.50, 0.30) after TNFi initiation, 0 (IQR -0.50, 0.40) after MTX initiation, and − 0.30 (IQR -1.0, 0.10) after metformin initiation. In unadjusted analysis, there was not a statistically significant difference in the median HbA1c change between TNFi and MTX. There was a statistically significant difference in the median HbA1c change between TNFi and metformin *(p < 0.001)* and between MTX and metformin (*p < 0.001)*. When compared to MTX initiators, TNFi initiators had a similar change in HbA1c in this expanded cohort, β 0.03 (95%CI: − 0.07, 0.14), *p = 0.552*. Metformin initiators had a significantly greater negative change in HbA1c than MTX initiators, β − 0.30 (95%CI: − 0.38, − 0.21), *p < 0.001* (Suppl Table S2, Suppl Figure 2).

We performed several sensitivity analyses to test assumptions made (Suppl Table S3, Suppl Table S4). First, we examined change in Hb1Ac by disease to determine whether there were differences for one particular disease group. The effect sizes were similar to the main results. After excluding patients with baseline glucocorticoid use, there was a smaller difference between TNFi and MTX initiators but results were similar in the metformin group. Similarly, adjusting for baseline TNFi exposure and allowing patients to only contribute one drug initiation did not differ from the results of the final model. We additionally repeated all of the analyses in the cohort of patients with diabetes but not restricted to HbA1c ≥ 7 (Suppl Table S5 and Suppl Table S6). Neither of the sensitivity analyses (adding calendar year to the model and restricting patients to a single therapy initiation) significantly changed the results.

## Discussion

In this retrospective cohort study in patients with DM and inflammatory arthritis, we found that, among patients with an elevated HbA1c at baseline, patients treated with TNFi and MTX had, on average, a small decrease in HbA1c that was approximately half of that seen with metformin initiation. We initially hypothesized, based on the mechanism of the TNFi, that TNFi would result in a greater decrease in HbA1c than MTX, but found that overall they were similar. These findings potentially support the concept that modulating inflammation associated with inflammatory arthritis may have off target benefits regardless of the therapy. We acknowledge the changes seen in this study were small and were not observed among all patients with diabetes when including patients with normal HbA1cs at baseline. Thus, additional studies are needed to confirm these results.

Few studies have evaluated the association between TNFi and their effect on fasting plasma glucose (FPG) and HbA1c values in inflammatory arthritis patients that also have a diagnosis of diabetes. In a six-month retrospective cohort study in psoriasis patients without diabetes treated with etanercept, infliximab, or methotrexate, there were no significant changes in FPG associated with any of the medications [45]. A 24-week study that evaluated nine non-diabetic psoriasis patients on etanercept did not find a significant difference in HbA1c and FPG values [46]. One small retrospective study assessing HbA1c, FPG, and fasting triglyceride levels in eight patients with RA or Crohn’s disease on etanercept or infliximab concluded that TNFi can improve glucose control in diabetic patients [47]. A randomized, double-blind study assessing diabetic patients with RA on etanercept versus a placebo control through week 12 and then an open label phase through week 24 found a slight decrease in FPG through week 12 and a slight decrease in HbA1c through week 24, but was not significant [48]. A retrospective study that evaluated a subgroup of patients with DM and RA (*n* = 75) found a significant decrease in HbA1c values after initiation of a TNFi [49]. Thus far, the findings above demonstrate an inconsistent effect of TNFi on HbA1c and/or FPG. To date, no studies have directly compared the effects of methotrexate versus TNFi on HbA1c values in patients with diabetes.

A handful of studies have also investigated change in HbA1c after treatment with a TNFi among patients without diabetes. One study assessed HbA1c and FPG values in psoriasis, PsA, or RA patients, the majority of which were non-diabetic, after initiation of etanercept, infliximab, adalimumab, or golimumab, versus methotrexate did not find a significant change in HbA1c or FPG [50]. A study of 39 AS patients and 18 PsA patients without diabetes did not demonstrate any change in mean FPG levels after initiating adalimumab, infliximab, or etanercept in the first 6 months of treatment [51]. Thus, both small studies found no significant benefit of TNFi on insulin resistance or HbA1c values in this patient population.

In designing this study, we focused primarily on patients with DM and with elevated HbA1c values as we felt that the off target effects of TNFi and MTX are of greater clinical importance in this population. In our study, we found greater improvements in HbA1c among the cohort with an elevated HbA1c at baseline than those with diabetes but not required to have an elevated baseline HbA1c. This likely is secondary to the fact that there was more room to change in the HbA1c ≥ 7 cohort; these patients were more likely to have uncontrolled DM at baseline. It is possible that HbA1c improved in all three treatment groups in this population due to regression to the mean. It was for this reason that we included the metformin group as a comparator and also examined all patients with diabetes. In the full diabetes population, the already small differences between MTX and TNFi were further attenuated, but overall, the results did not substantially change. Importantly, the effect was similar for both MTX and TNFi suggesting that they still had similar effects on HbA1c regardless of the patient population.

Potential limitations of the study include defining the diseases by diagnostic codes and limited knowledge of adherence to therapy (only prescriptions filled). Additionally, relatively long gaps between the baseline and follow-up HbA1c could have affected our results given the greater chance for factors such as medication changes, lifestyle (i.e., dietary) changes, and potential hospitalizations to affect HbA1c values. Gaps were similar between treatment arms including among the metformin group, however, which was included as a positive control. Similarly, patients with elevated HbA1c may have improved even without a therapy change. We did not examine patients not changing therapy in this study as it was not part of the original study question but may be one way to address the concern for regression to the mean. Next, we acknowledge that glucocorticoids can increase blood sugar. Accounting for glucocorticoid use is challenging given that it is likely on the causal pathway between treatment and HbA1c values. For this reason, we did not adjust for prednisone use nor account for prednisone use in time varying models. Similarly, use of diabetes medications were not accounted for in a time varying manner as they are on the causal pathway. We instead adjusted for use of diabetes medications at baseline. Next, in comparing methotrexate users to TNFi users, confounding by indication may exist (i.e., use of a TNF inhibitor may be associated with worse disease activity or longer disease duration, neither of which are measured in these datasets). We suspect that the confounding would have biased away from the null in the comparison of the two drugs; however, we found that TNFi and MTX had similar effects. Next, we selected a group of patients who had laboratory values available which are generally provided from two large laboratory vendors, likely requiring a certain type of insurance plan and that patients actually went to the lab to have their labs drawn. This may have caused a selection bias. We are unable to compare those with and without laboratory values as we only have patients with the lab values in the dataset. Finally, there was not a significant difference between TNFi and MTX. This could have been related to the relatively small sample size. However, there were enough patients available to identify a meaningful difference. Many of these limitations, including the need to examine the subset of the population with lab values, would be a limitation of any observational data source, but regardless, should be considered in interpretation of the findings.

Our study also has several strengths. We evaluated a large cohort of patients with RA, PsA, or AS with a diagnosis of DM. Optum contains one of the largest populations of RA, PsA, and AS patients and is a good representation of the United States insured population. Even after restricting to patients with lab values at the required time points, our sample size was relatively large. Finally, the use of metformin as a positive control provides internal and external validity to our study results.

## Conclusions

In conclusion, initiation of a TNFi or MTX among patients with an elevated HbA1c is associated with a modest decrease in HbA1c that is approximately half as much (**~** 0.4 units) as the decrease observed after initiation of metformin (~ 0.8 units). This study found no compelling evidence for a difference in the effect between TNFi and MTX, suggesting similar treatment effects. This study suggests that, in addition to limiting glucocorticoid exposure in patients with diabetes, controlling inflammatory disease may have off-target benefits, regardless of the drug choice. Future research is needed to understand the complex relationship between inflammatory arthritis, insulin resistance, glucocorticoids, and metabolic pathways.

## Supplementary information

**Additional file 1: Table S1.** Baseline Characteristics: Diabetes Mellitus Criteria Cohort*. **Table S2.** Associations Between Treatment Initiation and Change in HbA1c in Patients in the Diabetes Mellitus Criteria Cohort*. **Table S3.** Associations Between Treatment Initiation and Change in HbA1c in either PsA, RA, or AS Patients with Baseline HbA1c ≥ 7. **Table S4.** Sensitivity Analyses in the HbA1c >= 7 Cohort. **Table S5.** Associations Between Treatment Initiation and Change in HbA1c in either PsA, RA, or AS Patients in the Diabetes Mellitus Criteria Cohort*. **Table S6.** Sensitivity Analysis in the DM Criteria Cohort. **Figure S1.** Change in HbA1c in baseline A1c ≥ 7 cohort after TNFi, MTX, or Metformin Initiation. **Figure S2.** Change in HbA1c in DM Criteria Cohort after TNFi, MTX, or Metformin Initiation.

## Data Availability

Data can be obtained from Optum’s de-identified Clinformatics® Data Mart Database.

## Abbreviations

AS: Ankylosing spondylitis
BMI: Body mass index
CAD: Coronary artery disease
CHF: Congestive heart failure
CKD: Chronic kidney disease
COPD: Chronic obstructive pulmonary disease
CTD: Connective tissue disease
FPG: Fasting plasma glucose
HbA1c: Hemoglobin A1c
ICD: International classification of disease
MTX: Methotrexate
PsA: Psoriatic arthritis
RA: Rheumatoid arthritis
SMD: Standardized mean difference
TNFi: Tumor necrosis factor inhibitor
