# Supplementary material for: Impact of tumor necrosis factor inhibitors and methotrexate on diabetes mellitus among patients with inflammatory arthritis
Source: BMC Rheumatol. 2020 Sep 2;4:39. doi: 10.1186/s41927-020-00138-3 (PMC7466800; doi:10.1186/s41927-020-00138-3)
Supplement: Supplementary file 1 — Additional file 1: Table S1. Baseline Characteristics: Diabetes Mellitus Criteria Cohort*. Table S2. Associations Between Treatment Initiation and Change in HbA1c in Patients in the Diabetes Mellitus Criteria Cohort*. Table S3. Associations Between Treatment Initiation and Change in HbA1c in either PsA, RA, or AS Patients with Baseline HbA1c ≥ 7. Table S4. Sensitivity Analyses in the HbA1c >= 7 Cohort. Table S5. Associations Between Treatment Initiation and Change in HbA1c in either PsA, RA, or AS Patients in the Diabetes Mellitus Criteria Cohort*. Table S6. Sensitivity Analysis in the DM Criteria Cohort. Figure S1. Change in HbA1c in baseline A1c ≥ 7 cohort after TNFi, MTX, or Metformin Initiation. Figure S2. Change in HbA1c in DM Criteria Cohort after TNFi, MTX, or Metformin Initiation. [file 41927_2020_138_MOESM1_ESM.docx]

**Supplemental Table 1. Baseline Characteristics: Diabetes Mellitus Criteria Cohort***

|  | MTX  (N = 821) | TNFi  (N = 628) | Metformin (N = 3704) | SMD TNF vs MTX | SMD MTX vs Met |
| --- | --- | --- | --- | --- | --- |
| Mean age, years (SD)** | 45 (3) | 45 (3) | 45 (3) | 0 | 0 |
| Male sex (%)*** | 34% | 33% | 37% | -0.02 | 0.06 |
| Mean baseline HbA1c (SD) | 7.10 (1.4) | 7.02 (1.4) | 7.46 (1.5) | -0.06 | 0.32 |
| Baseline HbA1C≥7 (%) | 354 (43%) | 249 (40%) | 2,004 (54%) | -0.09 | -0.23 |
| Comorbidities, N (%) |  |  |  |  |  |
| Anemia | 139 (17%) | 107 (17%) | 448 (12%) | 0.00 | -0.35 |
| Angina | 128 (14%) | 90 (14%) | 424 (11%) | 0.00 | -0.25 |
| Anxiety | 116 (14%) | 102 (16%) | 718 (19%) | 0.14 | 0.31 |
| Asthma | 157 (19%) | 132 (21%) | 715 (19%) | 0.10 | 0.00 |
| CAD | 276 (34%) | 174 (28%) | 1010 (27%) | -0.20 | -0.23 |
| CHF | 115 (14%) | 67 (11%) | 430 (12%) | -0.25 | 0.16 |
| CKD | 222 (24%) | 120 (19%) | 628 (17%) | -0.24 | -0.34 |
| COPD | 134 (16%) | 109 (17%) | 716 (19%) | 0.06 | 0.18 |
| Cardiomyopathy | 43 (5%) | 16 (3%) | 131 (4%) | -0.55 | -0.25 |
| Other CTD | 54 (7%) | 34 (5%) | 138 (4%) | -0.36 | -0.58 |
| Depression | 205 (22%) | 178 (28%) | 957 (26%) | 0.24 | 0.17 |
| DM retinopathy | 108 (13%) | 76 (12%) | 49 (1%) | -0.08 | -1.36 |
| Hypertension | 723 (88%) | 524 (83%) | 3169 (86%) | -0.06 | -0.02 |
| Hypothyroidism | 307 (37%) | 230 (37%) | 1263 (34%) | 0.00 | -0.09 |
| Dyslipidemia | 735 (90%) | 560 (89%) | 3219 (87%) | -0.01 | -0.03 |
| Inflammatory bowel disease | 16 (2%) | 26 (4%) | 87 (2%) | 0.76 | 0.00 |
| Liver disease | 166 (20%) | 155 (25%) | 724 (20%) | 0.23 | 0.00 |
| Myocardial infarction | 49 (6%) | 27 (4%) | 58 (2%) | -0.44 | -1.00 |
| Obesity | 214 (26%) | 189 (30%) | 1116 (30%) | 0.15 | 0.15 |
| Psoriasis | 100 (12%) | 169 (27%) | 252 (7%) | 0.73 | -0.54 |
| Baseline diabetes medications, N (%) | 594 (72%) | 480 (76%) | 3704(100%) | 0.05 | 0.32 |
| Baseline MTX, N (%) | - | 328 (52%) | 465 (13%) | n/a | n/a |
| Baseline TNFi, N (%) | 79 (10%) | 206 (33%) | 316 (9%) | 0.96 | -0.11 |
| Baseline Steroids†, N (%) | 358 (44%) | 249 (40%) | 651 (18%) | -0.10 | -0.78 |
| Average baseline glucocorticoid dose, mean (SD) | 5 (6) | 5 (5) | 5 (6) | 0.00 | 0.00 |
| Duration btwn baseline and f/up HbA1c, mean # of days (SD) | 246 (86) | 243 (83) | 210 (82) | -0.04 | -0.44 |
| Duration btwn med start date and f/up HbA1c, mean # of days (SD) | 176 (68) | 172 (65) | 169 (66) | -0.06 | -0.11 |
| N = number of observations  * All patients with DM fulfilling criteria by ICD-9-CM codes and with a baseline HbA1c regardless of the value  ** In the metformin group, one observation is missing for age.  *** In the metformin group, three unknown observations for sex were changed to missing.  †Six-month baseline period  Abbreviations: CAD = coronary artery disease; CTD = connective tissue disease (ICD9 code 710.9, not inclusive of SLE, sicca, or scleroderma); CKD = chronic kidney disease; COPD = chronic obstructive pulmonary disease; CHF = congestive heart failure; DM = diabetes mellitus; HbA1c = hemoglobin A1c; MTX = methotrexate; SD = standard deviation; SMD = standardized mean difference; TNFi = tumor necrosis factor inhibitor. | | | | | |

**Supplemental Table 2. Associations Between Treatment Initiation and Change in HbA1c in Patients in the Diabetes Mellitus Criteria Cohort ***

| **Variable** | **Univariable Model** | | **Multivariable Model** | |
| --- | --- | --- | --- | --- |
|  | β^**^ | 95% CI | β^**^ | 95%CI |
| Treatment initiation |  |  |  |  |
| Methotrexate | Ref |  | Ref |  |
| TNFi | 0.08 | -0.03, 0.20 | 0.03 | -0.07, 0.14 |
| Metformin | -0.40 | -0.49, -0.30 | -0.30 | -0.38, -0.21 |
| Age (years) | 0.003 | -0.009, 0.02 | 0.006 | -0.004, 0.02 |
| Sex (female) | -0.17 | -0.25, -0.09 | -0.08 | -0.15, -0.02 |
| Baseline HbA1c | -0.53 | -0.56, -0.49 | -0.54 | -0.57, -0.50 |
| Baseline DM Medications | -0.13 | -0.26, -0.01 | 0.38 | 0.26, 0.49 |
| Atrial Fibrillation | 0.14 | 0.01, 0.26 |  |  |
| CAD | 0.13 | 0.05, 0.21 |  |  |
| CVD | 0.17 | 0.07, 0.26 |  |  |
| Dyslipidemia | 0.17 | 0.04, 0.29 |  |  |
| Hypothyroidism | 0.09 | 0.01, 0.16 |  |  |
| Liver Disease | -0.09 | -0.18, -0.003 | -0.12 | -0.19, -0.06 |
| Myocardial Infarction | 0.30 | 0.07, 0.52 | 0.26 | 0.03, 0.49 |
| PUD | 0.13 | 0.002, 0.27 |  |  |
| PVD | 0.14 | 0.03, 0.25 |  |  |
| ***** All patients with DM fulfilling criteria by ICD-9-CM codes and with a baseline HbA1c regardless of the value  **Beta-coefficients are interpreted as the mean difference in the outcome (HbA1c) in the target group (TNF or Metformin) minus the reference group (MTX).  Variables tested that were not significant at the univariable stage were not included in this table.  The following variables are not significant at the univariable stage: anemia, aortic aneurysm, angina, anxiety, asthma, baseline methotrexate, baseline TNFi, bipolar disorder, cancer, cardiomyopathy, coronary heart disease, congestive heart failure, chronic kidney disease, chronic obstructive pulmonary disease, connective tissue disease, degenerative disc disease, dementia, depression, diabetic retinopathy, giant cell arteritis, hypertension, inflammatory bowel disease, lung disease, mixed connective tissue disease, metabolic syndrome, obesity, obstructive sleep apnea, osteoarthritis, peripheral arterial disease, polymyalgia rheumatica, pregnancy, psoriasis, other psychiatric disorders, pulmonary embolism, rheumatoid arthritis-lung, SICCA syndrome, systemic lupus erythematous, sleep disorder, systemic sclerosis, and uveitis.  Abbreviations: CAD = coronary artery disease; CVD = cerebrovascular disease; DM = diabetes mellitus; HbA1c = hemoglobin A1c; PUD = peptic ulcer disease; PVD = peripheral vascular disease; TNFi = tumor necrosis factor inhibitor | | | | |

**Supplemental Table 3. Associations Between Treatment Initiation and Change in HbA1c in either PsA, RA, or AS Patients with Baseline HbA1c ≥ 7**

| **Variable** | **Multivariable Model (PsA)**  **N = 297** | | **Multivariable Model**  **(RA)**  **N = 1745** | | **Multivariable Model**  **(AS)**  **N = 717** | |
| --- | --- | --- | --- | --- | --- | --- |
|  | β | 95%CI | β | 95%CI | β | 95%CI |
| Treatment initiation |  |  |  |  |  |  |
| Methotrexate | Ref | - | Ref | - | Ref | - |
| TNFi | 0.25 | -0.14, 0.63 | 0.25 | -0.03, 0.52 | -0.16 | -0.98, 0.65 |
| Metformin | -0.40 | -0.75, -0.05 | -0.38 | -0.56, -0.21 | -0.34 | -0.89, 0.21 |
| Age (years) | 0.04 | 0, 0.08 | -0.004 | -0.02, 0.02 | -0.01 | -0.05, 0.02 |
| Sex (female) | -0.32 | -0.61, -0.03 | -0.20 | -0.34, -0.07 | -0.14 | -0.34, 0.06 |
| Baseline HbA1c | -0.59 | -0.73, -0.46 | -0.70 | -0.76, -0.63 | -0.67 | -0.76, -0.57 |
| Cardiomyopathy | -0.52 | -1.11, 0.07 | 0.36 | 0.01, 0.72 | 0.42 | -0.02, 0.87 |
| Other CTD | -0.38 | -0.94, 0.18 | -0.44 | -0.69, -0.19 | -0.40 | -0.97, 0.17 |
| Dyslipidemia | 0.12 | -0.31, 0.54 | 0.13 | -0.04, 0.29 | 0.25 | -0.07, 0.57 |
| Myocardial Infarction | 1.38 | 0.44, 2.33 | 0.16 | -0.26, 0.58 | 0.75 | -0.17, 1.66 |
| N = Number of observations  Abbreviations: AS = ankylosing spondylitis; CTD = connective tissue disease (ICD9 code 710.9, not inclusive of SLE, sicca, or scleroderma); HbA1c = hemoglobin A1c; PsA = psoriatic arthritis; RA = rheumatoid arthritis; TNFi = tumor necrosis factor inhibitor | | | | | | |

**Supplemental Table 4.** **Sensitivity Analyses in the HbA1c >= 7 Cohort**

| **Model** | **Comparison** | **Beta (95%CI)** |
| --- | --- | --- |
| Final Model | TNFi vs MTX | 0.22 (0.004, 0.43) |
|  | Met vs MTX | -0.38 (-0.52, -0.23) |
| Adjusting for baseline prednisone use (6 months) | TNFi vs MTX  Met vs MTX | 0.21 (-0.002, 0.42)  -0.42 (-0.57, -0.27) |
| Excluding baseline prednisone users (6 months) | TNFi vs MTX  Met vs MTX | 0.03 (-0.23, 0.30)  -0.47 (-0.68, -0.27) |
| Excluding baseline insulin users (6 months) | TNFi vs MTX  Met vs MTX | 0.45 (0.15, 0.74)  -0.16 (-0.33, 0.01) |
| Excluding insulin users in follow-up period | TNFi vs MTX  Met vs MTX | 0.38 (0.07, 0.69)  -0.18 (-0.37, 0.01) |
| Adjusting for baseline TNFi exposure (12 months) | TNFi vs MTX  Met vs MTX | 0.21 (-0.004, 0.43)  -0.38 (-0.52, -0.23) |
| Only allowing single exposure | TNFi vs MTX  Met vs MTX | 0.18 (-0.09, 0.45)  -0.45 (-0.61, -0.29) |
| Abbreviations: HbA1c = hemoglobin A1c; TNFi = tumor necrosis factor inhibitor; Met = metformin; MTX = methotrexate | | |

**Supplemental Table 5. Associations Between Treatment Initiation and Change in HbA1c in either PsA, RA, or AS Patients in the Diabetes Mellitus Criteria Cohort***

| **Variable** | **Multivariable Model (PsA)**  **N = 585** | | **Multivariable Model**  **(RA)**  **N = 3237** | | **Multivariable Model**  **(AS)**  **N = 1331** | |
| --- | --- | --- | --- | --- | --- | --- |
|  | β | 95%CI | β | 95%CI | β | 95%CI |
| Treatment initiation |  |  |  |  |  |  |
| Methotrexate | Ref | - | Ref | - | Ref | - |
| TNFi | 0.15 | -0.06, 0.35 | -0.02 | -0.16, 0.11 | 0.09 | -0.35, 0.52 |
| Metformin | -0.23 | -0.44, -0.02 | -0.31 | -0.42, -0.21 | -0.25 | -0.58, 0.09 |
| Age (years) | 0.03 | 0.01, 0.06 | 0.004 | -0.01, 0.02 | -0.005 | -0.02, 0.02 |
| Sex (female) | -0.10 | -0.26, 0.07 | -0.09 | -0.17, -0.01 | -0.06 | -0.18, 0.06 |
| Baseline HbA1c | -0.45 | -0.53, -0.37 | -0.54 | -0.59, -0.50 | -0.55 | -0.62, -0.48 |
| Baseline Diabetes Medications | 0.31 | 0.07, 0.54 | 0.37 | 0.23, 0.51 | 0.50 | 0.14, 0.85 |
| Liver Disease | -0.18 | -0.38, 0.02 | -0.15 | -0.24, -0.07 | -0.03 | -0.17, 0.11 |
| Myocardial Infarction | 0.45 | -0.24, 1.14 | 0.17 | -0.08, 0.43 | 0.39 | -0.19, 0.98 |
| N = Number of observations  ***** All patients with DM fulfilling criteria by ICD-9-CM codes and with a baseline HbA1c regardless of the value  Abbreviations: AS = ankylosing spondylitis; HbA1c = hemoglobin A1c; PsA = psoriatic arthritis; RA = rheumatoid arthritis; TNFi = tumor necrosis factor inhibitor | | | | | | |

**Supplemental Table 6. Sensitivity Analysis in the DM Criteria Cohort**

| **Model** | **Comparison** | **Beta (95%CI)** |
| --- | --- | --- |
| Final Model | TNFi vs MTX | 0.03 (-0.07, 0.14) |
|  | Met vs MTX | -0.30 (-0.38, -0.21) |
| Adjusting for baseline prednisone use (6 months) | TNFi vs MTX | 0.03 (-0.08, 0.14) |
|  | Met vs MTX | -0.32 (-0.41, -0.23) |
| Excluding baseline prednisone users (6 months) | TNFi vs MTX  Met vs MTX | -0.04 (-0.18, 0.10)  -0.34 (-0.46, -0.22) |
|  |  |  |
| Excluding baseline insulin users (6 months) | TNFi vs MTX  Met vs MTX | 0.08 (-0.04, 0.20)  -0.16 (-0.25, -0.07) |
| Excluding insulin users in follow-up period | TNFi vs MTX  Met vs MTX | 0.06 (-0.06, 0.18)  -0.16 (-0.26, -0.07) |
| Adjusting for baseline TNFi exposure (12 months) | TNFi vs MTX  Met vs MTX | 0.02 (-0.08, 0.13)  -0.30 (-0.38, -0.21) |
| Only allowing single exposure | TNFi vs MTX  Met vs MTX | 0.04 (-0.08, 0.15)  -0.28 (-0.37, -0.19) |
| Abbreviations: DM = diabetes mellitus; TNFi = tumor necrosis factor inhibitor; Met = metformin; MTX = methotrexate | | |

**
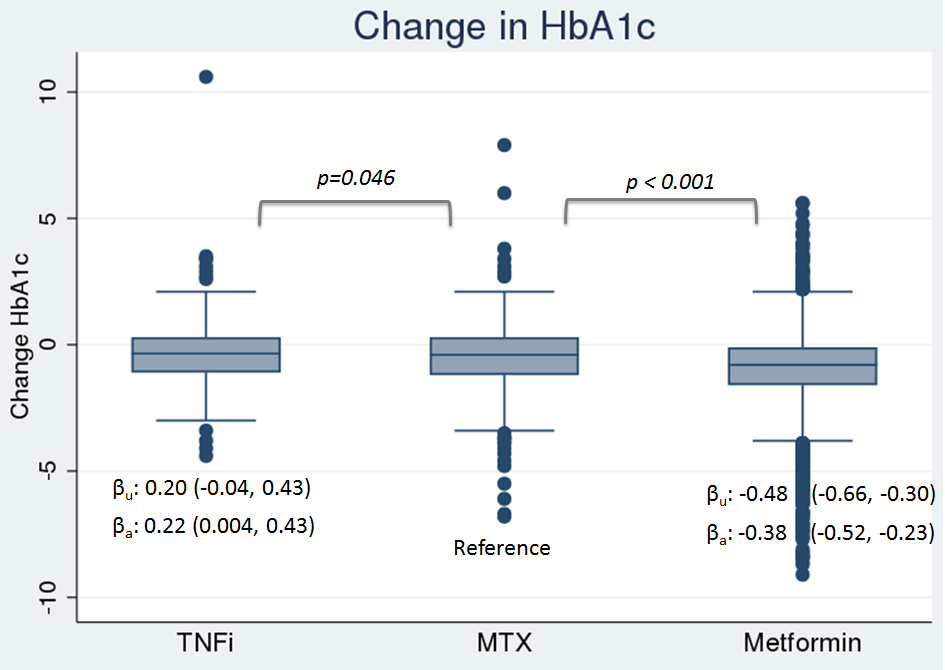
**

**Supplemental Figure 1. Change in HbA1c in baseline A1c ≥ 7 cohort after TNFi, MTX, or Metformin** **Initiation**.

β_u_: coefficient from unadjusted linear regression models; β_a_: coefficient from linear regression models adjusted for age, sex, baseline HbA1c, baseline DM medications, baseline MTX use (for patients on TNFi), and baseline comorbidities

Abbreviations: DM = diabetes mellitus; HbA1c = hemoglobin A1c; MTX = methotrexate; TNFi = tumor necrosis factor inhibitor

**
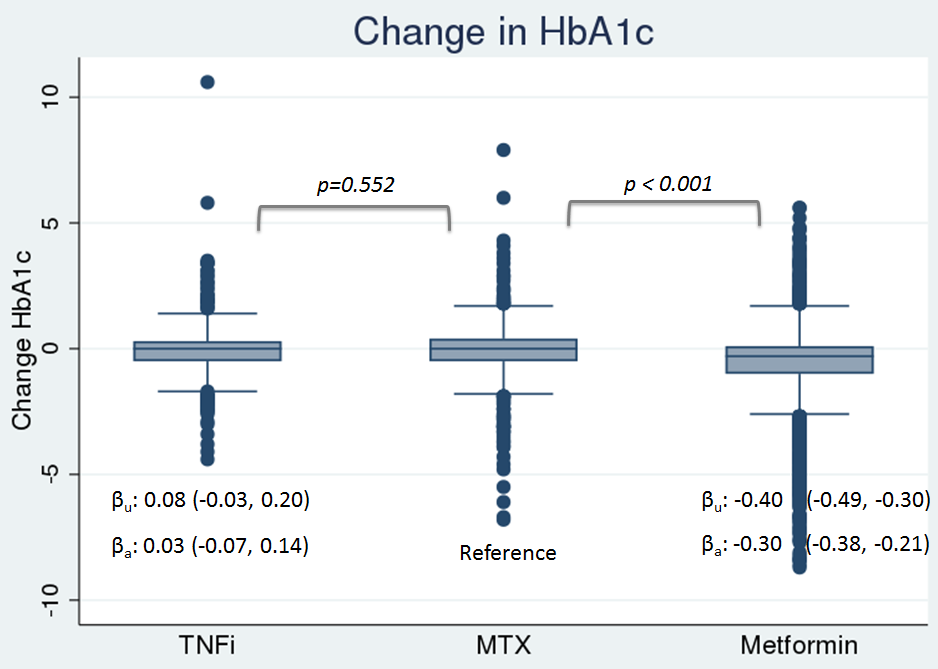
**

**Suppl Figure 2. Change in HbA1c in DM Criteria Cohort after TNFi, MTX, or Metformin Initiation.**

DM criteria cohort: all patients with DM fulfilling criteria by ICD-9-CM codes and with a baseline HbA1c regardless of the value. 51% of this cohort had a HbA1c≥7 at baseline.

β_u_: coefficient from unadjusted linear regression models; β_a_: coefficient from linear regression models adjusted for age, sex, baseline HbA1c, baseline DM medications, baseline MTX use (for patients on TNFi), and baseline comorbidities

Abbreviations: DM = diabetes mellitus; HbA1c = hemoglobin A1c; MTX = methotrexate; TNFi = tumor necrosis factor inhibitor
